# Planetary health education: a cornerstone for international sustainable public health deals

**DOI:** 10.1093/eurpub/ckac129.142

**Published:** 2022-10-25

**Authors:** C Cadeddu, J Pivor, M Jevtic, C Faerron, D Zjalic, GS Lombardi, SS Myers, W Ricciardi

**Affiliations:** 1 Department of Life Sciences and Public Health, Università Cattolica del Sacro Cuore, Rome, Italy; 2 Italian Institute for Planetary Health, Rome, Italy; 3 Planetary Health Alliance, Cambridge, USA; 4 Harvard TH Chan School of Public Health, Boston, USA; 5 Harvard University Center for the Environment, Cambridge, USA; 6 Faculty of Medicine, University of Novi Sad, Novi Sad, Serbia; 7 EUPHA-ENV

## Abstract

As stated in The Lancet Public Health editorial, “No public health without planetary health”, the future health of the planet and human health are inextricably linked. For this reason, global citizens, practitioners, and professionals, especially those involved in Public Health, must be equipped to address and understand the field of Planetary Health (PH), which looks at the complex connections associated with disruptions to natural systems and resulting impacts on human health. Strategies aimed at incorporating PH education into high schools and academic curricula are required to build capacity for future national and local PH leadership. One of the most relevant tools used to achieve these goals is the PH Education Framework, designed by the Planetary Health Alliance (PHA). This framework considers five foundational domains as essential for PH knowledge, values, and practice, and has been currently applied by different institutions involved in PH. The Italian Institute for PH (IIPH) applied this framework to a school-based project for education in urban health, which will be better described in the workshop presentation. The PH Education Framework domains were used for the development of four interactive sessions oriented to raise students’ interest on the topic and stimulate active participation during and after the intervention, also with simple pro-environmental behaviours. The experience was shown to be fruitful for Public Health residents as well, who were deeply involved in and led the interactive sessions held in the high school. By means of a qualitative assessment, residents demonstrated to have increased their self-confidence, knowledge and leadership skills in PH. Further research and applications of the PH Education Framework are needed in order to increase evidence and awareness in PH and strengthen PH collaboration in Europe and beyond.

